# Usefulness of Elastic Bandage Compression Compared to Calf Massage to Prevent Venous Thromboembolism—A Retrospective Evaluation

**DOI:** 10.3390/jcm13154355

**Published:** 2024-07-25

**Authors:** Keishi Kimura, Norio Imai, Asami Nozaki, Yoji Horigome, Hayato Suzuki, Hiroyuki Kawashima

**Affiliations:** 1 Department of Regenerative and Transplant Medicine, Division of Orthopedic Surgery, Niigata University Graduate School of Medical and Dental Science, Niigata 951-8510, Japan; kec.tnsfb.64@gmail.com (K.K.); inskawa@med.niigata-u.ac.jp (H.K.); 2 Division of Comprehensive Musculoskeletal Medicine, Niigata University Graduate School of Medical and Dental Sciences, Niigata 951-8520, Japan; hrgm070315@nifty.com

**Keywords:** prophylaxis, deep venous thrombosis, venous thromboembolism, total hip arthroplasty

## Abstract

**Background:** Manual calf massage and passive ankle motion (CaM) can reduce the incidence of venous thromboembolism (VTE) after total hip arthroplasty (THA). However, these methods cannot be used in all patients; thus, elastic bandage (EB) compression is an alternative method. The efficacy of EB compression in preventing VTE has not yet been investigated; thus, this study aimed to compare the effects of EB compression and manual calf massage. **Methods:** Of the 363 patients who underwent unilateral primary THA at our hospital between 1 August 2018 and 31 October 2023, CaM without anticoagulation therapy was administered to 206 patients (CaM group) and 157 patients underwent EB without anticoagulation therapy (EB group). Duplex ultrasonography was performed 7 days postoperatively to check for deep vein thrombosis (DVT) in both legs. **Results:** The surgical time (122.2 min vs. 155.5 min), the incidence of DVT (4.5% vs. 4.8%) and pulmonary thromboembolism (PTE) (0% vs. 0.7%), intraoperative bleeding (305.4 mL vs. 301 mL), and estimated actual blood loss (846.6 mL vs. 811.6 mL) were not significantly different between the CaM and EB group. However, there was one case of symptomatic PTE in the EB group. **Conclusions:** The incidences of DVT, PTE, and intraoperative bleeding were not significantly different between the groups. Moreover, EB can be administered to patients with DVT and is considered to be a DVT prophylaxis method that can be used in a larger number of patients. Therefore, we recommend that EB be performed in all the patients undergoing THA.

## 1. Introduction

Venous thromboembolism (VTE) is one of the crucial complications after total hip arthroplasty (THA) [1]. VTE is a prevalent medical condition with high morbidity, mortality, and associated costs, and patients may develop post-thrombotic syndrome [2,3]. Various prophylactic interventions for VTE have been recommended [4,5,6], and mechanical and chemical preventative measures, such as foot pumps, calf pumps, and anticoagulation therapy, are frequently performed. Several studies have reported that the absolute risk of VTE in Japanese patients is similar to that in European and North American patients [7,8]. Greets et al. showed that the incidence of deep vein thrombosis (DVT) after THA without antithrombotic drugs is 42–57%, and the morbidity due to pulmonary thromboembolism (PTE) is 0.1–1.2% [9]. Around 90% of the symptomatic PTE is generated from DVT [10]; hence, DVT prophylaxis after THA is crucial in order to prevent symptomatic and critical PTE.

We previously demonstrated that manual calf massage and passive ankle motion (CaM) after THA can reduce the incidence of DVT [11]. CaM is a quick passive exercise for the dorsoplantar flexion of the ankle joint, while massaging is performed on the thickest part of the calf after THA. We also reported that the incidences of DVT and PTE were not significantly different between the CaM with anticoagulation therapy and CaM-only groups. However, perioperative blood loss differed between the groups and increased in the CaM with anticoagulation therapy group. Therefore, we recommended that CaM be performed as a preventive measure, and that postoperative anticoagulant therapy is not required for routine use in inpatients without DVT [12]. Because of the methods of CaM, the operator (surgeon or assistant in our hospital) who performed the CaM was tired. CaM is potentially undesirable in terms of anesthesia management because the body of the patient is shaken during CaM, and the patient may awaken if the anesthesia does not have sufficient effect, or in rare cases, laryngoconvulsions may occur. Moreover, it is contraindicated for patients with DVT to receive intermittent pneumatic compression, a venous foot pump [13], and mechanical stimulation, such as CaM; therefore, this method may not be applicable to all patients.

Elastic bandage (EB) compression has been reported as a method for DVT prevention and treatment [14]. Several randomized open-label European trials have reported a 50% relative reduction in the risk of PTE with the use of EB compression for 2 years after DVT [15]. The American College of Chest Physicians Evidence-Based Clinical Practice Guidelines recommend 30–40 mmHg of ankle compression pressure for DVT prophylaxis [16]. Therefore, we hypothesized that intraoperative compression with an EB could be used for perioperative VTE prophylaxis. This study aimed to compare the efficacy of EB compression in preventing perioperative VTE with that of CaM as the usefulness of EB compression in preventing THA-related VTE. We hypothesized that the prevention of VTE using EB is equal to that of using CaM, even without the administration of anticoagulation agents.

## 2. Materials and Methods

The study was conducted in accordance with the Declaration of Helsinki and approved by the Institutional Review Board of Niigata University Graduate School of Medical and Dental Sciences (2021-0023), and this study was registered on 4 August 2021. Informed consent for the study was obtained from all the participants.

**Figure 1:**
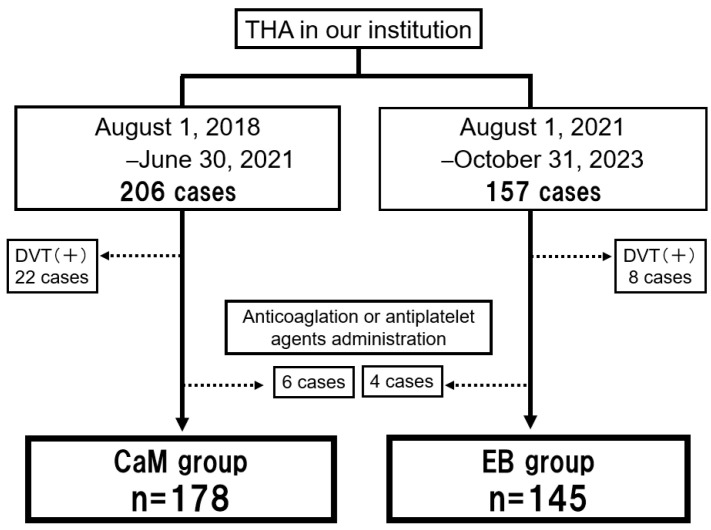
Patient selection flowchart. THA, total hip arthroplasty; DVT, deep vein thrombosis; CaM, manual calf massage and passive ankle motion; EB, elastic bandage.

We retrospectively reviewed 363 patients who underwent unilateral primary THA using the same surgical approach (anterolateral supine approach), and pre- and postoperative protocols at our hospital between 1 August 2018 and 31 October 2023. A total of 206 patients received CaM without anticoagulation therapy (CaM group) between 1 August 2018, and 30 June 2021, and 157 patients received EB on their calves (EB group) between 1 August 2021 and 31 October 2022. We excluded the patients who underwent bilateral THAs on the same day and hip surgery, such as revision THA and pelvic or femoral osteotomy. All the patients underwent preoperative duplex ultrasound to observe the veins in both legs. Patients on antithrombotic drugs for VTE and those with a history of cerebrovascular or cardiac disease were excluded. Consequently, 28 and 12 patients were excluded from the CaM and EB groups, respectively (Figure 1). A total of 323 patients were included in the final analyses.

All the operations were performed using an anterolateral approach, with the patient placed in a supine position under general anesthesia. Cementless cups and stems were used in the study. We administered 1 g tranexamic acid intravenously 10 min before surgery and 6 h after the first administration to reduce perioperative bleeding [17].

In the CaM group, CaM was performed after THA according to the methods described in previous studies [9,10]. CaM was performed 30 times on the operating table for 10 s immediately after THA. The hip joint was positioned at 30° of flexion and the knee was fully extended. The legs were then lowered for 10 s to restore venous circulation. This procedure was repeated three times on both legs and completed within about 1 min. An intermittent pneumatic compression device was applied to the calf of the non-operative leg before the surgery and after the induction of general anesthesia, and on the calf of both legs after surgery, until the first postoperative day in both groups. Compression stockings were worn on both legs for 10 d postoperatively.

**Figure 2:**
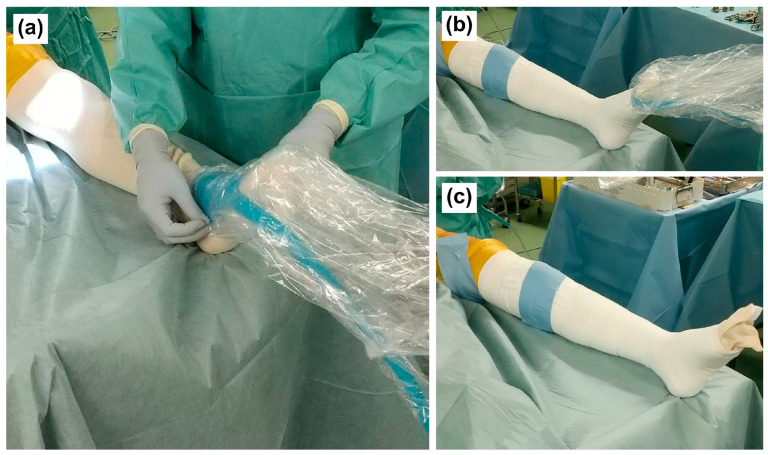
How to wrap the EB for the EB group before surgery. (**a**) A pressure gauge that was covered with sterile vinyl was placed on the anterior aspect of the ankle joint; (**b**) an EB was wrapped from the foot to the thigh of the patient so that the pressure is 34–43 mmHg; (**c**) the pressure gauge was then removed. EB, elastic bandage compression.

For the EB group, a pressure gauge (Palm Q ^®^, CAPE, Yokosuka, Japan) was placed on the anterior aspect of the ankle joint on the surgical side, and the gauge was wrapped with an EB before surgery (Figure 2a,b). In the same way as a previous study [18], the pressure gauge was removed after ensuring that the intraoperative compression pressure was 30–40 mmHg and that the measured value was 34–43 mmHg (Figure 2c).

In both groups, the patients were allowed to leave their beds, fully weight-bearing, on the first postoperative day, and anticoagulation therapy was not applied for postoperative thromboprophylaxis. The patients were administered celecoxib (400 mg) for 7 days postoperatively for pain control. Duplex ultrasonography was performed 7 days postoperatively to check for DVT in both legs. A skilled vascular technician performed the exam and an experienced radiologist reviewed the results. All of them were blinded to the group classifications of the patients. In addition, we performed a blood examination to determine the hemoglobin concentration, platelet count, prothrombin time (PT)-international normalized ratio (INR), and activated partial thromboplastin time (aPTT) at 1 and 7 days postoperatively. We set the standard for patient discharge with a single cane from the hospital to their homes at approximately 10 days after THA. The outcomes in this study included the patients’ age, gender, operation time, intraoperative bleeding, and total perioperative estimated blood loss following the formula of Gross [19] and Nadler [20]:

estimated actual blood loss (eABL) = estimated blood volume (mL) (hematocrit reduction/mean hematocrit).

Hematocrit reduction was defined as the difference between the preoperative hematocrit value and the hematocrit value 7 days postoperatively. The incidence of DVT was assessed using duplex ultrasonography 7 days postoperatively, and the incidence of PTE was compared retrospectively.

We examined the platelet counts, PT, PT-INR, and aPTT for the purpose of determining the coagulability before THA in the two groups.

We used the SPSS statistical software (ver. 28; IBM Corp., Armonk, NY, USA) for the statistical analysis. For comparing quantitative data, Student’s *t*-test was used after confirming normality with the Shapiro–Wilk test. Regarding the comparison of qualitative data, the chi-square test was used for data such as the number of male and female patients and the operative side, while Fisher’s exact test was used when the expected value of the cell in the 2 × 2 contingency table was less than 10. Outlier tests were not conducted because the outliers were not considered to be caused by obvious errors. Statistical significance was set at *p* < 0.05. Moreover, for a multivariate regression analysis, the study subjects were grouped into one group. The presence or absence of VTE was an independent variable, and dependent variables were defined for factors for which *p* < 0.2 was obtained in the univariate analysis and CaM or EB.

## 3. Results

The details of the participants are presented in Table 1. Multivariate analysis was performed with aPTT, operative time, CaM, and EB, which were *p* < 0.2 in univariate analysis, as the dependent variables, and with or without VTE as the independent variables, but no factors were statistically significant (Table 1 and Table 2). There were no significant differences in age, sex, side, body mass index, platelet counts, aPTT, PT, and PT-NR between the groups (Table 1). Moreover, surgical time, the incidence of DVT and PTE, intraoperative bleeding, and eABL were not significantly different between the groups (surgical time, 122.2 ± 40.8 min vs. 155.5 ± 31.9 min; DVT, 4.5% vs. 4.8%, all of them were asymptomatic; PTE, 0% vs. 0.7%; intraoperative bleeding, 305.4 ± 172.8 mL vs. 301 ± 161.5 mL; total blood loss, 846.6 ± 270.5 mL vs. 811.6 ± 257.5 mL) (Table 2). However, there was one case of symptomatic PTE with low oxygen saturation and without dyspnea in the EB group detected on day 4 after THA.

## 4. Discussion

We investigated whether EB is as effective as CaM in preventing DVT in the absence of anticoagulation agents. The incidences of VTE and intraoperative bleeding were not significantly different between the EB and CaM groups. This indicated that EB is as effective and safe as CaM for thromboprophylaxis. We previously reported that CaM with anticoagulant therapy reduces the incidence of DVT and has comparable perioperative blood loss compared to only anticoagulant therapy [11], and compared CaM and CaM with anticoagulation and found no difference in the incidence of DVT or PTE, demonstrating the usefulness of CaM. Furthermore, the disadvantages of CaM, such as operator fatigue and the risk of waking the patient up from anesthesia due to the shaking of the body, have been resolved by the EB procedure, which involves wrapping the EB around the leg before surgery. In contrast to foot pumps, calf pumps, and CaM, EB can be performed in patients with DVT, and the fact that it does not exacerbate DVT is an advantage.

Previous reports have shown a symptomatic PTE rate of 0.21–0.4% and have recommended the aggressive use of anticoagulant therapy [21,22,23]. They also reported that physical prophylaxis and the use of anticoagulants have gradually decreased DVT, but the incidence of PTE has not changed significantly [22]. In this study, one case of symptomatic PTE occurred in the EB group, but the difference was not significant compared to the CaM group, and the incidence of PTE in this study was comparable to that reported by other authors.

The initial goal of the VTE treatment is to slow the formation of active thrombi and prevent the formation of new thrombi while allowing the thrombolytic process to proceed and restore/maintain venous blood flow [24]. EB decreases interstitial effusion, resulting in improved tissue perfusion, which may play an anti-inflammatory role [25]. In addition, the compression of the superficial veins may increase deep blood flow velocity and prevent thrombus formation by decreasing the venous cross-sectional area. Moreover, EB assists the pump function of the leg muscles, which may prevent thrombus formation by inhibiting vein wall tortuosity associated with muscle pump failure and preventing venous valve dysfunction [25,26,27,28,29]. From these points of view, the EB technique can be used in patients with thrombosis and may reduce the incidence of perioperative DVT.

This study had some limitations. First, this was not a randomized study, but a serial series study. All the patients were selected from a single institution at a university hospital. However, the patients were not selected based on their preoperative group affiliation, which reduced selection bias. Second, it is unclear whether intraoperative ankle joint pressure was maintained at 30–40 mmHg. Third, we performed duplex ultrasonography only once preoperatively and postoperatively. However, all the patients were followed up at the University Hospital outpatient clinic and did not complain of calf pain or edema, which are suggestive of DVT, or the signs of pulmonary embolism. Moreover, duplex ultrasonography was performed to detect DVT, but contrast venography is more sensitive than ultrasound for detecting DVT [30]. However, contrast venography is invasive and, therefore, impractical as a method of repeat testing. In contrast, duplex ultrasonography is non-invasive, safe, and repeatable. Therefore, this method has become standard for DVT diagnosis in most hospitals in Japan. Finally, we could not directly compare the group with no prevention for VTE, including physical prophylaxis, and the EB group to examine the effectiveness of EB, because it is ethically difficult to compare the EB method with no physical prophylaxis under various guidelines in recent years.

## 5. Conclusions

The incidences of DVT, PTE, and intraoperative bleeding were not significantly different between the groups. Moreover, EB can be administered to patients with DVT and is considered a DVT prophylaxis method that can be used in a larger number of patients.

Therefore, we recommend that EB be performed in all the patients undergoing THA. This study was able to demonstrate the thromboprophylactic effect of the EB method by measuring pressure; however, not all facilities have pressure sensors. Therefore, methods for EB wrapping with appropriate pressure need to be explored in the future.

## Figures and Tables

**Table 1:** Preoperative data for each group.

	CaM Group (*n* = 178)	EB Group (*n* = 145)	*p*-Value
Age (years) ^1^	61.6 ± 12.1	62.2 ± 12.8	0.667 ^2^
Sex (men/women)	51/127	32/113	0.349 ^3^
Right/left	95/83	76/69	0.924 ^3^
BMI (kg/m^2^) ^1^	22.9 ± 3.8	24.0 ± 3.7	0.662 ^2^
Plt (×10^4^) ^1^	25.1 ± 6.7	24.2 ± 6.9	0.269 ^2^
aPTT (second) ^1^	30.1 ± 3.2	30.7 ± 3.0	0.167 ^2^
PT (%) ^1^	108.3 ± 11.2	109.0 ± 10.4	0.633 ^2^
PT-INR ^1^	1.0 ± 0.1	1.0 ± 0.1	1.921 ^2^
Reasons for surgery			
DDH	161	132	0.994 ^3^
ONFH	14	11	
Others	3	2	

^1^ mean ± standard deviation. ^2^ *t*-test. ^3^ chi-square test. No significant difference in patient background between CaM and EB. CaM: manual calf massage and passive ankle motion; EB, elastic bandage compression; BMI, body mass index; Plt, platelet count; aPTT, activated partial thromboplastin time; PT, prothrombin time; INR, international normalized ratio.

**Table 2:** The incidence and rate of VTE.

	CaM Group (*n* = 178)	EB Group (*n* = 145)	*p*-Value
Surgical time (min)	122.2 ± 40.8	115.5 ± 31.9	0.196 ^2^
Intraoperative blood loss (g)	305.4 ± 172.8	301 ± 161.5	0.224 ^2^
Total blood loss (mL)	846.6 ± 270.5	811.6 ± 257.5	0.237 ^2^
DVT cases (%)	8 (4.5%)	7 (4.8%)	0.896 ^3^
PTE cases (%)	0	1 (0.7%) ^1^	0.267 ^3^

^1^ This case is included in the list of DVT cases. ^2^ *t*-test. ^3^ Fisher exact test. CaM: manual calf massage and passive ankle motion; EB, elastic bandage compression; DVT, deep vein thrombosis; PTE, pulmonary thromboembolism.

## Data Availability

The original contributions presented in the study are included in the article, further inquiries can be directed to the corresponding author.
